# Why Quorum Sensing Controls Private Goods

**DOI:** 10.3389/fmicb.2017.00885

**Published:** 2017-05-19

**Authors:** Martin Schuster, D. Joseph Sexton, Burkhard A. Hense

**Affiliations:** ^1^Department of Microbiology, Oregon State UniversityCorvallis, OR, United States; ^2^Institute of Computational Biology, Helmholtz Zentrum MünchenNeuherberg, Germany

**Keywords:** quorum sensing, cooperation, cheating, public good, private good, evolutionary stability, *pseudomonas aeruginosa*, nucleoside hydrolase

## Abstract

Cell-cell communication, also termed quorum sensing (QS), is a widespread process that coordinates gene expression in bacterial populations. The generally accepted view is that QS optimizes the cell density-dependent benefit attained from cooperative behaviors, often in the form of secreted products referred to as “public goods.” This view is challenged by an increasing number of cell-associated products or “private goods” reported to be under QS-control for which a collective benefit is not apparent. A prominent example is nucleoside hydrolase from *Pseudomonas aeruginosa*, a periplasmic enzyme that catabolizes adenosine. Several recent studies have shown that private goods can function to stabilize cooperation by co-regulated public goods, seemingly explaining their control by QS. Here we argue that this property is a by-product of selection for other benefits rather than an adaptation. Emphasizing ecophysiological context, we propose alternative explanations for the QS control of private goods. We suggest that the benefit attained from private goods is associated with high cell density, either because a relevant ecological condition correlates with density, or because the private good is, directly or indirectly, involved in cooperative behavior. Our analysis helps guide a systems approach to QS, with implications for antivirulence drug design and synthetic biology.

## Introduction

Cell-cell communication or quorum sensing (QS) is a prevalent mechanism that coordinates gene expression in bacterial populations via diffusible chemical signals (Waters and Bassler, 2005; West et al., 2007a; Schuster et al., 2013; Hense and Schuster, 2015; Popat et al., 2015). These self-produced signal molecules accumulate with increasing cell density and trigger gene expression by activating a cognate receptor. A common theme is that QS primarily controls collective, cooperative traits such as biofilm formation, virulence, social motility, and nutrient digestion. Most of these group activities involve the secretion of extracellular products generally referred to as “public goods,” including exopolysaccharides, toxins, surfactants, and extracellular enzymes. At the transcriptome level, the response to QS-signaling can be dramatic, with hundreds of genes (about 5% of the genome) controlled by QS in the opportunistic pathogen *Pseudomonas aeruginosa* (Hentzer et al., 2003; Schuster et al., 2003; Wagner et al., 2003). Here, too, the set of genes encoding secreted factors is overrepresented compared to the genome at large (Gilbert et al., 2009). A similar overrepresentation of secretion is observed in the QS regulons of *Vibrio* and *Erwinia* species (Antunes et al., 2007; Barnard et al., 2007).

**Figure 1:**
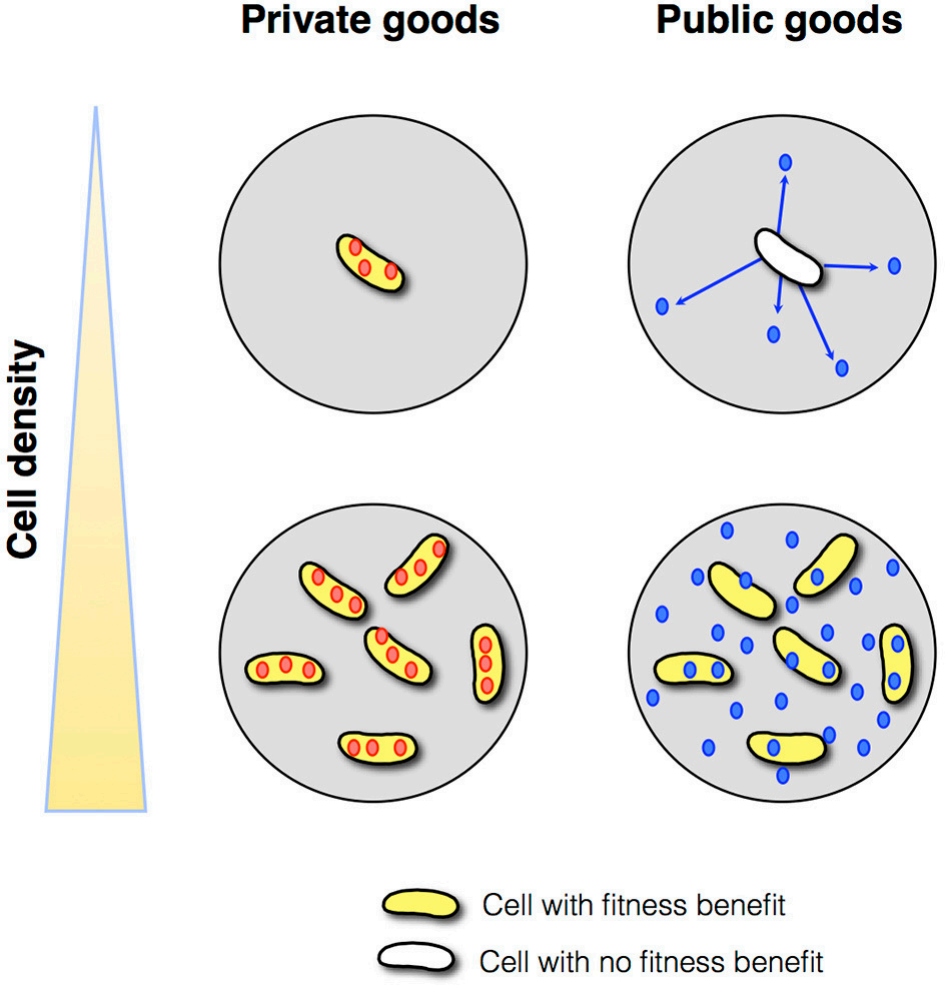
**Private vs. public goods**. Assuming appropriate environmental conditions, cell-associated, private goods (red circles) provide a benefit to the producing cell irrespective of cell density. In contrast, diffusible public goods (blue circles) provide a benefit to the producing cell at high density but not at low density.

Theoretical arguments show that the benefit from a secreted public good usually increases with population density, justifying regulation by QS (Pai and You, 2009; Heilmann et al., 2015; Popat et al., 2015). At low density, a large fraction of the public good is lost to the environment by mass transfer, whereas at high density, environmental losses are reduced because the product benefits neighboring cells (Figure 1). These considerations are supported by a small number of empirical studies that show a positive density-dependent benefit for QS-controlled public goods, in one case a protease in *P. aeruginosa* (Darch et al., 2012), and in another case an antibiotic-degrading enzyme in a synthetic QS system (Pai et al., 2012).

**Figure 2:**
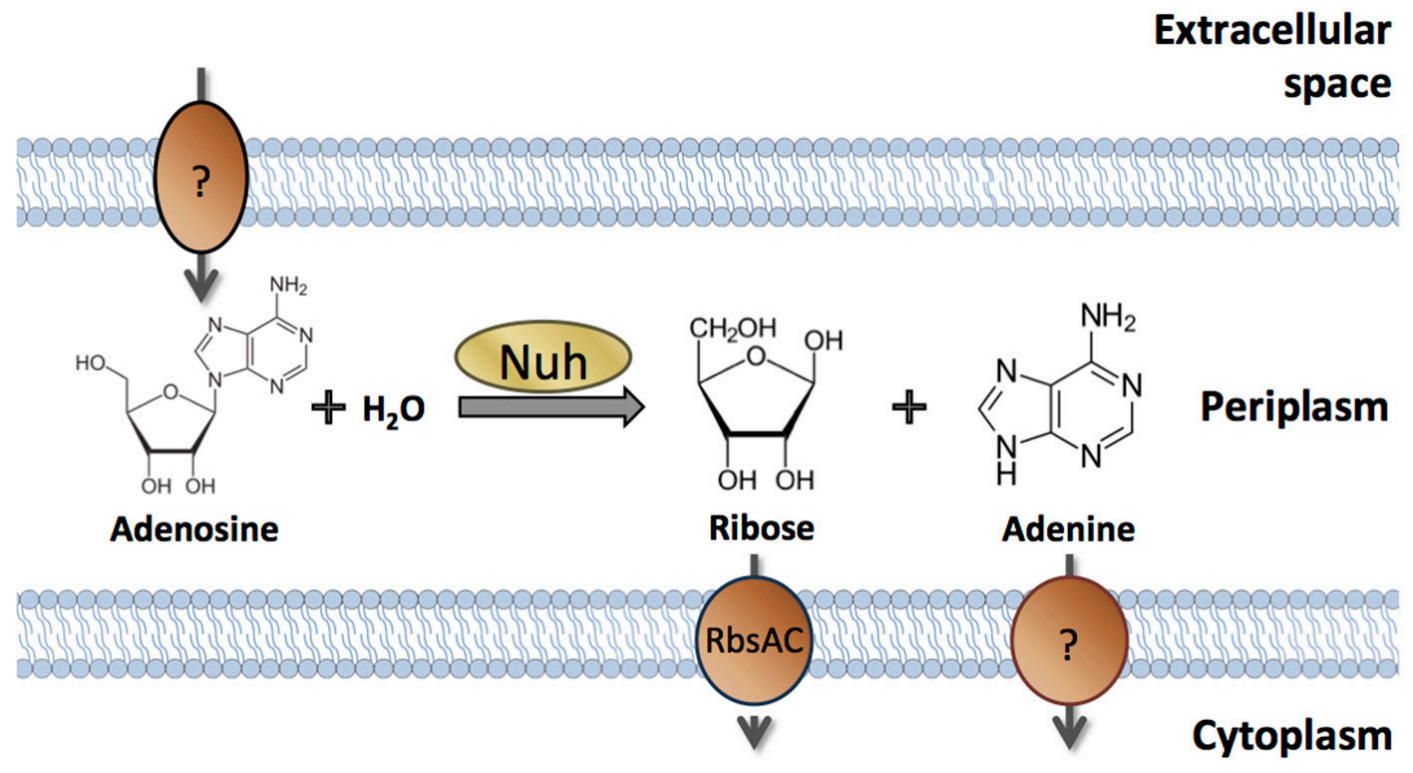
**Cellular location and enzymatic activity of ***P. aeruginosa*** Nuh**. Periplasmic Nuh produces ribose and adenine from the hydrolysis of adenosine. Inosine may serve as an alternative substrate that is converted into ribose and hypoxanthine (not shown). Specific transporters take up the products into the cytoplasm. The proteins involved in nucleoside transport across the outer membrane (if any) and in adenine nucleobase transport across the inner membrane have not been identified in *P. aeruginosa*.

Nevertheless, there are numerous examples of cell-associated products that are QS-controlled (Table 1). These products can be proteins or metabolites; they can be intracellular (cytoplasmic) or surface-attached. They have been designated private goods because their location or benefit is assumed to be exclusive to the producing cell (Figure 1). Perhaps the most prominent QS-controlled private good is *P. aeruginosa* nucleoside hydrolase (Nuh) (Heurlier et al., 2005; Mellbye and Schuster, 2011; Dandekar et al., 2012; Darch et al., 2012). It is a periplasmic enzyme that hydrolyzes adenosine and inosine, allowing the cell to grow on these nucleosides as the sole carbon (C) or nitrogen (N) source (Heurlier et al., 2005; Figure 2). For many cell-associated traits, it is not immediately clear why they are under the control of QS. *A priori*, their benefit appears non-collective and hence, should be independent of cell density.

**Table 1:** **Cell-associated factors involved in QS-controlled behaviors^**a**^**.

**Behavior/phenotype**	**Cell-associated factor**	**Species**
**ANTICIPATION OF STRESS AT HIGH CELL DENSITY**		
Oxidative stress resistance	Catalase, superoxide dismutase, dehydrogenase	*P. aeruginosa*
Antibiotic resistance	Aminoglycoside acetyltransferase	*P. stewartii*
Type VI secretion	Immunity proteins	*B. thailandensis*
Phage resistance	Phage receptor OmpK, CRISPR-Cas immunity	*V. anguillarum*,
		*P. aeruginosa*
Competence	Competence proteins	*B. subtilis*
Sporulation	Sporulation proteins	*B. subtilis*
Metabolic slowing	Enzymes involved in glucose uptake, glycolysis, oxidative phosphorylation, nucleotide metabolism	*B. glumae*
Alternative carbon catabolism	Maltose-fermentation and glyoxylate-cycle enzymes	*Y. pestis*
Biofilm dispersal	Surfactant putisolvin, flagella	*P. putida*
**METABOLIC CHANGES ASSOCIATED WITH SECRETION**		
Type II secretion	Xcp secretory apparatus	*P. aeruginosa*
Type II secretion	Secretory apparatus	*B. cepacia*
Cyanide production	Hydrogen cyanide synthase	*P. aeruginosa*
Rhamnolipid secretion	Rhamnosyl transferase	*P. aeruginosa*
Cps biosynthesis	Exopolysaccharide	*P. stewartii*
Cyanide resistance	Alternative cytochrome c oxidase	*P. aeruginosa*
AHL metabolism	Serine hydroxymethyltransferases, glycine cleavage system proteins	*P. aeruginosa*
Adenosine, inosine catabolism	Nucleoside hydrolase (Nuh)	*P. aeruginosa*
Carbon compound catabolism	Cytochrome oxidases from oxidative phosphorylation, Entner-Doudoroff pathway enzymes	*P. aeruginosa*
**LEAKY FUNCTIONS WITH PUBLIC AND PRIVATE BENEFITS**		
Oxidative stress resistance	Catalase, superoxide dismutase	*P. aeruginosa*
Acetate switch	Acetyl-CoA synthase	*V. fischeri*
Antibiotic resistance	Aminoglycoside acetyltransferase	*P. stewartii*
Fructose catabolism	Invertase	Yeast
Iron acquisition	Siderophores	*E. coli*
		*P. aerginosa*
Adenosine, inosine catabolism	Leaking periplasmic Nuh or its metabolites	*P. aeruginosa*

a *We list behaviors and associated factors (e.g., in the form of proteins) according to the classification and the examples cited in the main text. Some behaviors fit into more than one class*.

In an attempt to explain the QS-control of private goods, some considered a potential function in the stabilization of co-regulated cooperative behaviors by public goods (Dandekar et al., 2012; Oslizlo et al., 2014; Majerczyk et al., 2016). The evolution and maintenance of cooperative behaviors is difficult to explain because they are subject to exploitation by cheaters that reap the benefits of cooperation without paying the costs (Hamilton, 1964; Hardin, 1968; Lehmann and Keller, 2006; West et al., 2007b). For example, in *P. aeruginosa*, QS-deficient cheater mutants can invade a population of QS-proficient cells that produce an extracellular protease required for growth on a proteinaceous C-source (Diggle et al., 2007; Sandoz et al., 2007; Dandekar et al., 2012; Pollitt et al., 2014; Asfahl et al., 2015). When the Nuh-dependent substrate adenosine is present as an additional C-source, however, the evolution of protease-deficient cheaters is constrained (Dandekar et al., 2012). This and other examples have emphasized the role of QS-regulated private goods in cheater control (Foster et al., 2004; Wilder et al., 2011; Dandekar et al., 2012; Oslizlo et al., 2014; Garcia-Contreras et al., 2015; Wang et al., 2015). In this article, we highlight the difference between cheater control by private goods as an adaptation (its “evolutionary purpose”) and as a by-product of selection for other benefits. Based on physiological and ecological considerations, we suggest several alternative functions for the QS control of private goods that all entail the optimization of a cell-density dependent net benefit. This benefit is realized either because there is a favoring ecological condition that correlates with high cell density, or because the private good itself is, directly or indirectly, linked to cooperative behavior. We discuss the implications of our analysis for the control of infections with novel antivirulence drugs and for the engineering of stable cooperative circuits in synthetic biology and biotechnology.

## Did the QS-control of private goods evolve to constrain cheating?

**Figure 3:**
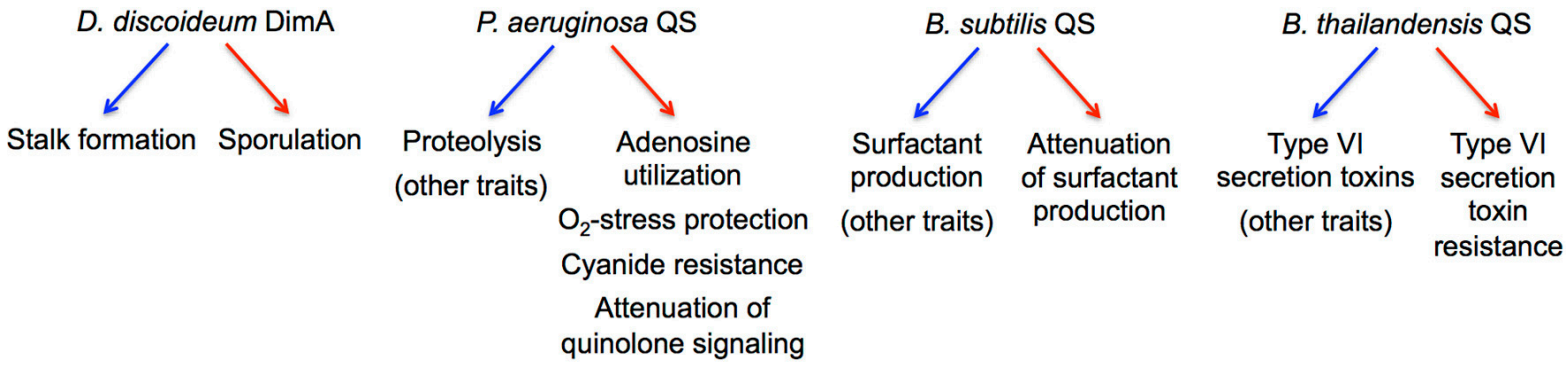
**Microbial examples of pleiotropic regulation implicated in cheater control**. The regulation of cooperative and non-cooperative behaviors in the respective species is indicated by blue and red arrows, respectively.

The maintenance of cooperation through co-regulation of multiple traits, generally referred to as pleiotropic constraint, has been initially described in the fruiting-body-forming amoeba *Dictyostelium discoideum* (Foster et al., 2004; Figure 3). The principal idea is that pleiotropic constraint stabilizes cooperation by linking the expression of a cooperative, public good to a non-cooperative, private good, often through common regulation. Private good production provides a benefit to a producer but not to a cheater in which common regulation is disrupted.

Several QS-controlled private goods have been investigated in this context (Figure 3). In addition to Nuh (Heurlier et al., 2005), these include catalase and superoxide dismutase in *P. aeruginosa* that detoxify reactive oxygen species (Hassett et al., 1999), an alternative cytochrome *c* oxidase in *P. aeruginosa* that provides resistance to self-produced hydrogen cyanide (Cunningham et al., 1997), and immunity proteins that protect against contact-dependent inhibition by Type VI secretion effectors in *B. thailandensis* (Majerczyk et al., 2016). Consequently, the growth advantage of QS-deficient cheaters in a cooperative environment decreases when private good expression is favored, i.e., in the presence of adenosine, oxidative stress, cyanide production, or Type VI-proficient cells (Dandekar et al., 2012; Garcia-Contreras et al., 2015; Wang et al., 2015; Majerczyk et al., 2016). Furthermore, the pleiotropic, QS-controlled suppression of costly functions in *P. aeruginosa* and *Bacillus subtilis* can also restrict the spread of certain QS-deficient mutants in co-culture (Wilder et al., 2011; Oslizlo et al., 2014). In all these cases, co-regulation of private goods appears to stabilize cooperation under specific culture conditions, but fundamentally, it is not clear whether these forms of cheater control represent an adaptation or a by-product of selection for other benefits.

**Figure 4:**
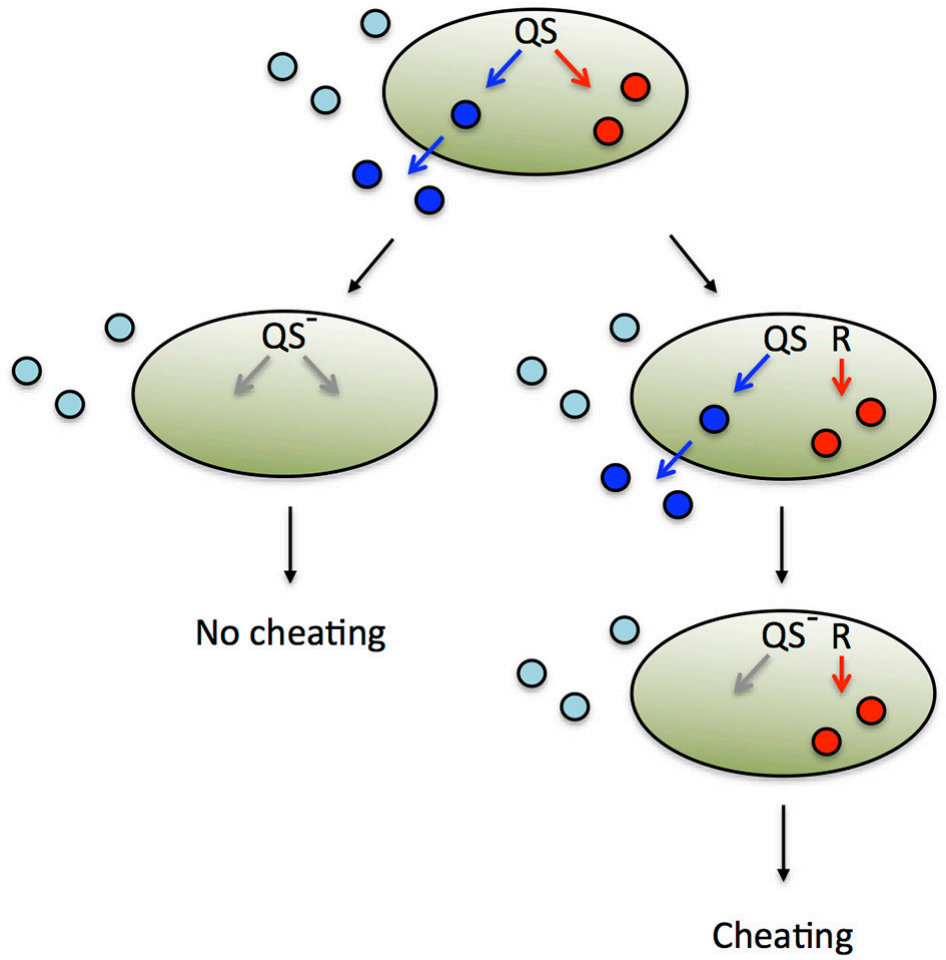
**Alternative regulatory schemes and evolutionary fates**. The expression of secreted, public goods (blue) and private goods (red) is co-regulated by QS (top). Public goods produced by the focal cell (dark blue) are distinguished from those produced by other cells (light blue). A loss-of function mutation in a QS pathway component (generally a transcriptional regulator) renders a strain deficient in both public and private good expression (left). In an environment that favors private good expression, this strain is unable to cheat and exploit public good production by the group. However, this form of pleiotropic cheater control is not evolutionarily stable, because it is susceptible to invasion by an alternative strategy (right). One or multiple mutational events can render private good expression independent of QS. In the example shown, private good expression is brought under the control of a different regulator or regulatory system, R. When a successive mutation in QS renders the strain deficient in public good expression, it is able to cheat and spread, because private good expression is now independently regulated.

To examine this question, we consider the concept of an evolutionarily stable strategy (ESS) derived from evolutionary game theory (see Box 1). Individual strategies are assigned a fitness pay-off or net benefit and compared. Net benefit is defined as the difference between benefit and cost. An ESS is a strategy that is resistant to invasion by an initially rare, alternative strategy. Thus, in order to determine whether the QS control of private goods for the purpose of cheater control is an ESS, we must consider alternative strategies. One such strategy is the QS-independent expression of the private good, uncoupled from common QS regulation (Figure 4). While QS-controlled expression of the private good disfavors cheaters, constitutive expression or no expression at all may confer a substantial net benefit to the producer under certain conditions. This is apparent if we consider the fact that QS restricts gene expression to a high cell density environment conducive to cooperation. For instance, when private good production is beneficial at low density, then constitutive expression invades QS-dependent expression. On the other hand, when private good production is beneficial neither at low nor high density, then no expression invades QS-dependent expression, as it saves costly private good production. The potential benefit from QS-independent expression can be further optimized if the expression of the private good is brought under the control of a dedicated regulatory system (Figure 4). Then the private good can be expressed only if it provides a benefit, irrespective of cell density.

Box 1 **Game-theoretical approach to cheater control by pleiotropic constraint.**

Game theoretical approaches have a long history in social evolution, and they formally define the concept of an evolutionarily stable strategy (ESS), a strategy that cannot be invaded by an alternative strategy (Smith and Price, 1973; Maynard Smith, 1982; Sigmund, 2011). One particular game, the Prisoner's dilemma, encapsulates the problem with cooperation, including the sharing of public goods (Tucker, 1950). Two players each choose whether to cooperate with the other player or to defect (cheat). Cooperation provides the greatest average advantage but unilateral defection provides the greatest individual advantage. In a version of this game that separately considers costs and benefits, a cooperator pays a cost *c* for another individual to receive a benefit *b* > *c*. A defector, in contrast, does not provide benefits and incurs no costs, but can still benefit from cooperation. Costs and benefits from pairwise interactions are defined in a pay-off matrix (figure insets). Based on these pay-offs, we model population dynamics using the replicator equation, a rate equation for the relative sizes of subpopulations (Taylor and Jonker, 1978; Schuster and Sigmund, 1983; Asfahl and Schuster, 2016). Initial frequencies of cooperator and defector subpopulations are 50:50, 90:10, and 10:90. As is well established, the strategy “defect” is an ESS and dominates the strategy “cooperate” **(A)**, because the pay-off from defection is always higher than the payoff from cooperation, in encounters with cooperators as well as with defectors (i.e., *b* > *b*-*c* and 0 > -*c*).

We employ the intuitive and well-established Prisoner's dilemma here to illustrate basic ESS thinking, although more complex frameworks exist (Archetti and Scheuring, 2012). We also do not consider the effects of spatial structure, consistent with experimental studies on this topic (Dandekar et al., 2012; Oslizlo et al., 2014; Garcia-Contreras et al., 2015; Wang et al., 2015). This is reasonable if one assumes that cheater control mechanisms likely have a role in stabilizing cooperation when kin selection or assortment, primarily achieved through population structuring in microbes, is less effective.

As a next step, we consider pleiotropic constraint as a cooperation-stabilizing principle, specifically the common regulation of public and private goods by QS. We term this strategy linked cooperation. Private good production confers specific benefits and costs that are exclusive to the producer. When regarding private benefits and costs as independent of social interactions and thus remaining constant, we can express the difference between them as net benefit, for short. Thus, our pay-off matrix is modified such that cooperation increases the pay-off by a net benefit *q*, in interaction with both cooperators and defectors. When we model population dynamics according to the modified pay-off matrix, we find that cooperation is indeed stabilized if the benefit *q* from the QS-controlled private behavior exceeds the cost of cooperation *c* **(B)**.

We now consider an alternative strategy that renders private good production independent of QS. As explained in the main text, independent expression can increase fitness if, for example, expression is directly tied to the available benefit. A mutant with this strategy thus gains a net benefit q^*^ that is higher than *q* for the original strategy (unlinked cooperation vs. linked cooperation, respectively). It is obvious, then, that the alternative strategy is able to invade the original strategy **(C)**. With public and private good control uncoupled, the invader is now itself susceptible to invasion by a defector mutant, essentially recreating the original Prisoner's dilemma described above **(A)**.

Taken together, pleiotropic regulation for the purpose of cheater control cannot be considered an ESS because it can be invaded by alternative strategies. This conclusion is consistent with other studies on the evolutionary stability of cheater control mechanisms. These studies focused primarily on coercive behaviors such as policing in social insects (Frank, 1995; Queller, 2006; Gardner et al., 2007; Lehmann et al., 2007; Ratnieks and Wenseleers, 2008). As social behaviors, they underlie the same evolutionary constraints as cooperation itself. The key point is, then, that cheater control mechanisms only represent solutions to the problem of cooperation if they are capable of evolving by natural selection.

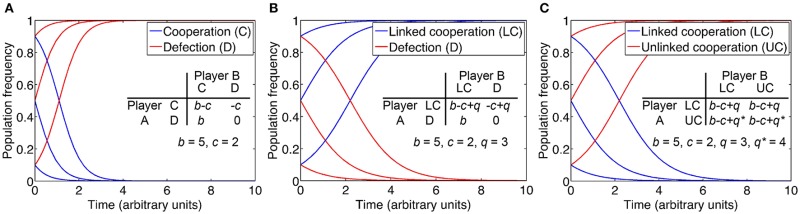

These considerations challenge the notion that the QS-control of private goods has evolved and is maintained for the purpose of cheater control. Indeed, dynamic modeling shows that such pleiotropically constrained cooperation is susceptible to invasion by mutant strategies that independently express the private good (Box 1). Once the joint regulation of both public and private goods is interrupted, a defector can invade and cooperation is no longer stable. Consequently, QS-controlled private good expression can only be an ESS if it provides a direct fitness advantage that is superior to QS-independent expression. We propose several functions below that may satisfy this requirement.

## What is a private good? mechanism informs function

To formulate insightful alternative hypotheses about the function and adaptive significance of private goods, a detailed mechanistic understanding is essential. We consider biochemical, physiological and ecological context. Importantly, demonstrating that a particular mechanism is well suited to a specific laboratory environment is different from claiming that this mechanism was selected for in the past due to its history of being adaptive. Thus, *in vitro* culturing experiments constitute a double-edged sword. They provide valuable insights into possible adaptations to specific selection pressures, but it is often not clear whether these selection pressures are relevant in the natural environment (Bailey and Bataillon, 2016).

By a common economical definition, private goods are both excludable and rivalrous, meaning that their benefits are only available to the producer, and that consumption by one necessarily prevents consumption by another (Kaul and Mendoza, 2003). In the microbial realm, a distinction between location and benefit is important, as is the appropriate designation of the relevant component involved in the behavior. Suppose an intracellular enzymatic pathway and its secreted metabolic endproduct; for example, the biosynthetic enzymes that produce exopolysaccharide (Vu et al., 2009). Here, the enzymes are the private goods, and the metabolic end-product is the public good. However, what we are interested in is not the enzymes *per se* but rather the benefit that the metabolic endproduct confers. Thus, the relevant good with respect to the resulting cooperative behavior is the extracellular metabolite. Because the enzyme is directly involved in the production of the metabolite, its connection to cooperative behavior and hence, regulation by QS, is obvious. As we will see below, this link may not be as obvious in other instances, such as central metabolic enzymes that help synthesize precursors of a secreted metabolite.

Once the relevant good has been identified, its classification as a strictly private or strictly public good may not always be clear-cut. Is a private good really exclusive to the producing cell or is partially shared by others? Economists typically call such goods with both public and private characteristics “mixed goods” (Pearce, 2002), whereby the balance between public and private benefits can vary. A mixed good that is partially shared by others can provide a cell-density-dependent benefit and would thus justify regulation by QS. The interplay between public and private aspects can also have remarkable effects on population dynamics and evolutionary stability. In a striking example from yeast, the products generated by a cell-associated enzyme, invertase, largely diffuse away; only 1% is captured by the producing cell (Gore et al., 2009). Yet, this small fraction is sufficient to allow a rare invertase-proficient strain to invade an invertase-negative cheater population. On the other hand, the density-dependent, cooperative nature of the activity also allows a rare cheater to invade an invertase-proficient population, resulting in an equilibrium of cheaters and co-operators (Gore et al., 2009).

Taken together, inferences about possible regulatory needs require a comprehensive mechanistic understanding of the phenotype in question which, as we will see below, is not always straightforward.

## The case of Nuh

The QS-controlled private good that has arguably received the greatest attention is *P. aeruginosa* Nuh (Heurlier et al., 2005; Mellbye and Schuster, 2011; Dandekar et al., 2012; Darch et al., 2012). Nucleoside hydrolases are part of the nucleotide salvage pathway (Nyhan, 2014). This pathway permits the recycling of nucleosides and bases generated during the degradation of DNA and RNA, from both intracellular and extracellular sources. *P. aeruginosa* Nuh also promotes growth on adenosine and inosine as the sole C or N sources (Heurlier et al., 2005). Because QS induces Nuh expression about five-fold (Schuster et al., 2003), the growth of a QS-deficient mutant is impaired on these nucleosides (Heurlier et al., 2005). Nuh cleaves adenosine into adenine and ribose, and inosine into hypoxanthine and ribose (Heurlier et al., 2005, 2006; Figure 2). Adenine is a good N but a poor C-source in *P. aeruginosa*, although its immediate metabolic fate and possible conversion to hypoxanthine are not entirely clear (Heurlier et al., 2006). Hypoxanthine is degraded to glyoxylate and urea in several steps (Matsumoto et al., 1978). Glyoxylate can be converted to acetyl-CoA, which is oxidized in the TCA cycle to yield cellular energy and C-building blocks (Schuster et al., 2003; Heurlier et al., 2005). Ribose can be converted to ribose-5-phosphate by ribokinase for anabolic and catabolic reactions via the pentose phosphate pathway (Stover et al., 2000). We assume for now that these Nuh metabolites remain intracellular and that the treatment of Nuh as a private good is meaningful. We will examine this assumption in the next section.

Interestingly, Nuh is periplasmic in *P. aeruginosa*, whereas the three homologs in in *E. coli* are cytoplasmic (Petersen and Moller, 2001; Imperi et al., 2009a). The periplasmic location in *P. aeruginosa* might be a consequence of the poor nucleoside transport across the cytoplasmic membrane that lacks known nucleoside importers (West, 1996; Stover et al., 2000). In contrast to other Pseudomonads, *P. aeruginosa* also seems impaired in ribose uptake across the cytoplasmic membrane. It lacks the *rbsD* gene that has been shown to contribute to uptake (Oh et al., 1999). This property explains the slow growth rates on adenosine as the sole C-source (doubling times ~10 h) (Heurlier et al., 2005; Mellbye and Schuster, 2011; Dandekar et al., 2012) and indicates that adenosine, via ribose, does not serve as an ecologically relevant C-source in *P. aeruginosa*. It is instead the adenine moiety of adenosine that *P. aeruginosa* seems to be after, as an N-source or as a direct precursor for nucleic acid synthesis. Consistent with this notion, prolonged N-starvation increases Nuh activity (Fields, 2002; dissertation data not published elsewhere). The regulatory mechanism and its relationship to QS, however, are not clear. Regulation of *nuh* by the N-starvation sigma factor RpoN was proposed by Fields et al. but could not be confirmed experimentally (Schulz et al., 2015).

These mechanistic insights permit differentiated hypotheses about relevant ecophysiological context. Extracellular adenosine and other nucleosides are likely to be abundant when the environment contains damaged cells such as during infection or competition with other microbes (Patel et al., 2007; Gellatly and Hancock, 2013). These conditions could be a direct consequence of QS-induced secretion at high cell density: Nuh is part of a broad array of QS-controlled factors, including many extracellular enzymes and toxins, that can damage other cells to release nutrients. Here, Nuh would scavenge nucleosides for nucleic acid biosynthesis and as general N source. This property could then help control cheating by QS mutants, even though it may not have evolved for this purpose. A *lasR* mutant would have a growth disadvantage because it would not be able to utilize nucleosides to the same extent as the wild-type. This QS-specific effect might increase or decrease with N-limitation, depending on regulatory interactions.

**Figure 5:**
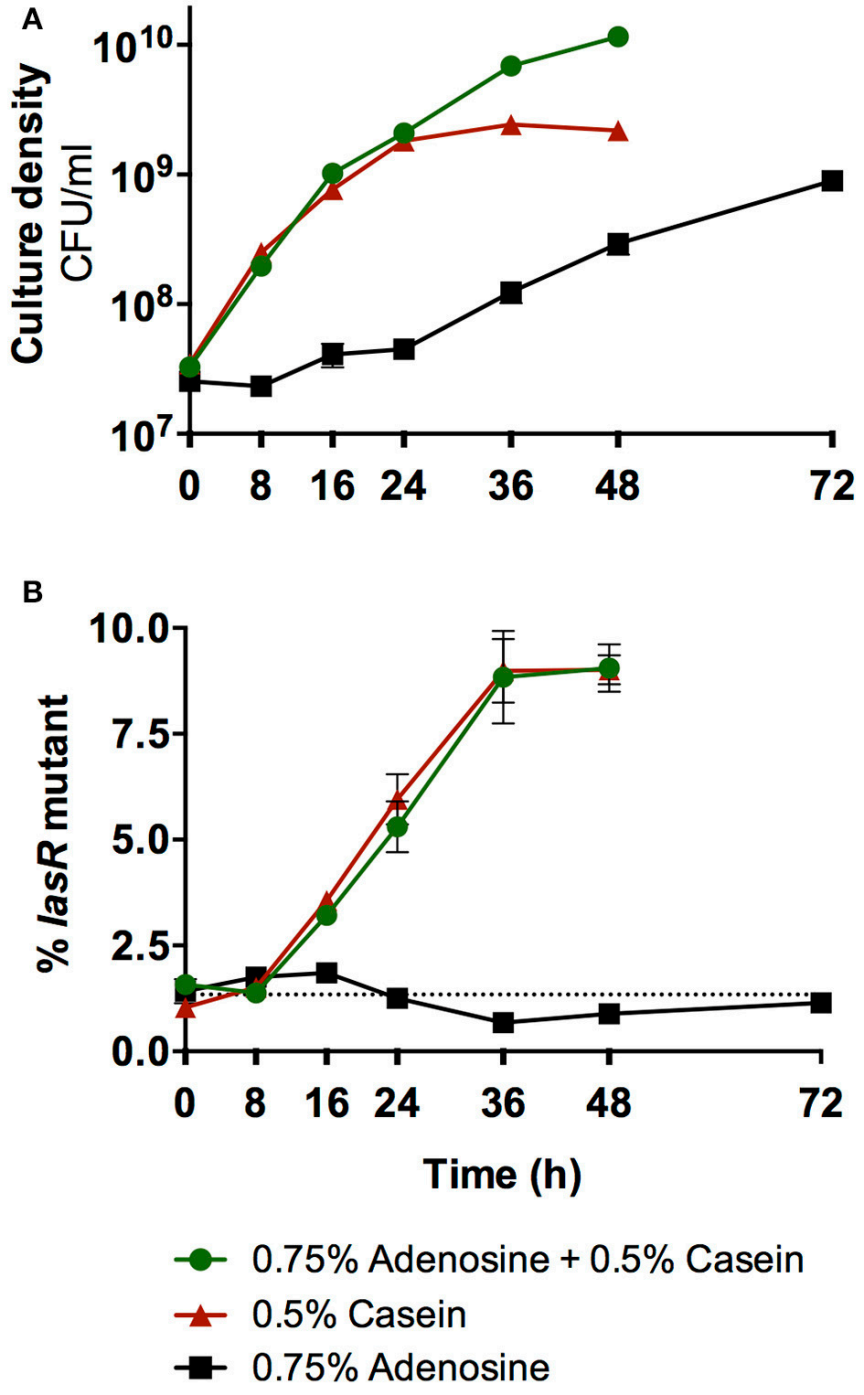
**Effect of adenosine catabolism on QS cheater control**. **(A)** Total growth of co-cultures of the *P. aeruginosa* PAO1 wild-type and the isogenic *lasR* deletion mutant. **(B)** Enrichment of the *lasR* mutant in co-culture. Co-cultures were initiated at ~1% mutant frequency in minimal medium with either casein, adenosine, or casein and adenosine as sole C-sources, modeled after Dandekar et al. (2012). Cultures were incubated at 37°C with shaking. Total growth and enrichment of the *lasR* mutant were quantified by plate counts. The *lasR* mutant contained a trimethoprim resistance marker at a neutral chromosomal site that allowed differentiation from the wild-type (Wilder et al., 2011). The presence of the marker does not affect growth (Wilder et al., 2011). The dotted line indicates the starting mutant frequency as an average of all three conditions. Error bars indicate standard deviation of the mean of three experimental replicates and are too small to be visible in some instances.

In contrast, when selection is for adenosine as the C-source, as in Dandekar et al. (2012), then adaptations are predicted to be entirely different. Dandekar et al. reported that the evolution of *lasR* mutant cheaters in wild-type populations is suppressed when adenosine is provided in the growth medium along with casein as the sole C-sources (Dandekar et al., 2012). This outcome was attributed to pleiotropic control of both secreted proteases and cell-associated Nuh by QS. However, alternative interpretations are possible. Dandekar et al. only observed cheater control at very high concentrations of adenosine, when we expect Nuh activity to be maximal and subsequent cellular uptake to be rate-limiting. Under these conditions, QS-dependent differences in Nuh expression should have little effect on adenosine utilization and consequently on cheater control. To test this prediction empirically, we initiated co-cultures of the *P. aeruginosa* wild-type and a defined *lasR* mutant in medium containing either casein, adenosine, or both as the sole the C-sources, at concentrations equal to Dandekar et al. (Figure 5). A critical difference from Dandekar et al. is that these experiments were conducted with defined strains such that effects from undefined mutations that may arise during *in vitro* evolution of an all-wild-type population are excluded. We found that casein medium alone favors *lasR* mutant cheaters, as previously reported (Sandoz et al., 2007; Dandekar et al., 2012). In support of our predictions, we also found that adenosine medium alone does not favor either strain, and that the addition of adenosine to casein medium has no effect on *lasR* mutant enrichment compared with casein medium alone.

Given these results, how was the evolution of *lasR* mutants nevertheless suppressed in the original study? A second, intriguing observation provides insights: Successive rounds of subculturing selected for mutants with greatly improved growth on adenosine (Dandekar et al., 2012). These variants are expected to harbor mutations that increase ribose uptake, given that this function is impaired in *P. aeruginosa*. Such non-social adaptation to nutrient-limited environments has been reported in several studies (Morgan et al., 2012; Waite and Shou, 2012; Asfahl et al., 2015). Thus, *P. aeruginosa* may have entirely shifted C-source preferences from casein to the high levels of available adenosine that sustained growth throughout the growth cycle. Taken together, non-social adaptation to the specific growth environment, rather than pleiotropic control of cooperative growth, may have lead to the observed suppression of *lasR* mutants.

## Explanations for the QS-control of private goods

**Figure 6:**
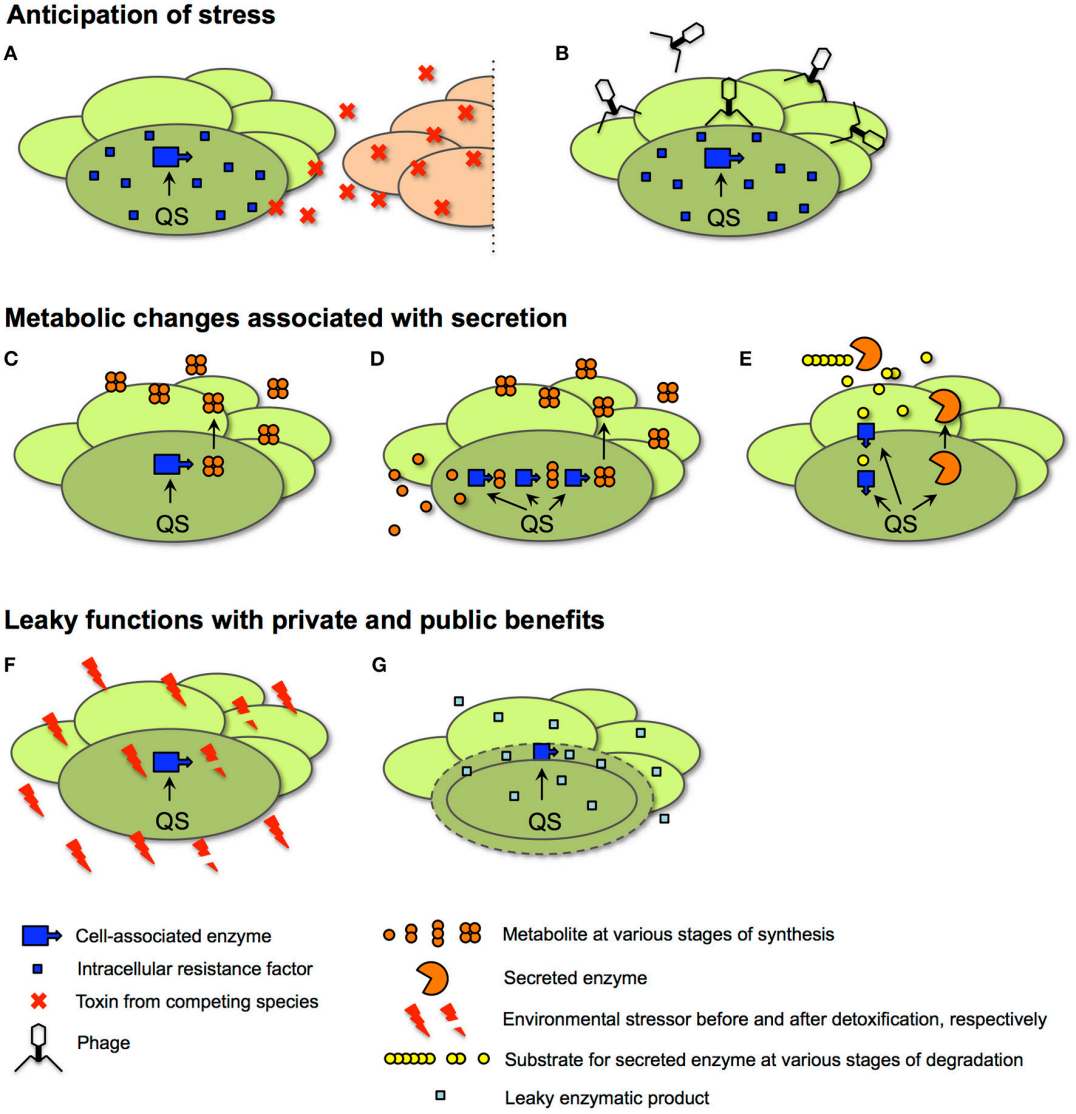
**Scenarios for the QS control of private goods**. In all cases, details of intracellular processes are shown for only one representative cell (darker shade of green) of a bacterial population at high cell density. **(A,B)** Anticipation of stress. In the examples shown, an intracellular resistance factor produced by a QS-controlled enzyme anticipates the need for protection from a toxin secreted by a competing species (red ovals in **A**) or from infection by phage **(B)**. **(C–E)** Metabolic changes associated with secretion. In an example for a direct link to secretion **(C)**, QS activates an intracellular enzyme responsible for the synthesis of a secreted metabolite. In a more indirect contribution to secretion, QS adjusts central metabolism **(D)**. Here, intracellular enzymes provide precursors for the production of the secreted metabolite. In a third metabolic function **(E)**, QS co-regulates enzymes involved in the uptake and intracellular processing of a degradation product generated by a secreted enzyme. The secreted metabolite and the secreted enzyme are examples of public goods. **(F,G)** Leaky functions with public and private benefits. In an example of homeostatic control of the shared environment **(F)**, an environmental stressor such as reactive oxygen species or acid is detoxified by an intracellular enzyme. In an example of periplasmic leakage **(G)**, the products of a periplasmic enzyme leak into the extracellular space; only a fraction is retained by the producing cell.

In the following section we categorize and discuss several plausible scenarios that explain the QS-dependent regulation of private goods (summarized in Table 1 and Figure 6). We consider relevant physiological and ecological context. In all cases, there is a cell-density dependent benefit to the expression of private goods, even though it is often not as obvious as that attained from public goods. Thus, these scenarios are in line with the principle that QS optimizes the net benefit attained from a product under its control (Pai and You, 2009). The benefit from the QS control of private goods is realized either because there is a specific ecological condition, generally a stressor, that is associated with high cell density, or because the private good itself is, directly or indirectly, associated with cooperation.

### Anticipation of environmental conditions: a stress response to high cell density

Populations at high cell density often face the effects of crowding: Accumulation of waste products, nutrient exhaustion, and competition with others (Navarro Llorens et al., 2010; Cornforth and Foster, 2013). Dedicated signaling pathways exist to respond to these biotic stressors. For example, responses to slow growth and nutrient exhaustion include the general stress response mediated by the alternative sigma factor RpoS and the stringent response mediated by the alarmone ppGpp (Battesti et al., 2011; Hauryliuk et al., 2015). These and other responses have mainly been associated with intracellular protection, but the secretion of toxins, including bacteriocins and antibiotics, appears equally important (Cornforth and Foster, 2013). Such actions directed at competing cells suggest that stress responses detect and respond to ecological competition by other cells, a concept recently introduced as competition sensing (Cornforth and Foster, 2013; Figure 6A). A focal cell infers the presence of competitors directly from cell damage or indirectly via nutrient limitation.

Importantly, if cell density is a reliable predictor of the specific stress condition, then QS could be beneficial as the sole regulatory input (Goo et al., 2012; Cornforth and Foster, 2013). QS may be able to infer stress earlier than a dedicated signaling pathway that directly detects the condition. A significant fitness benefit from early anticipation, paired with the predictive power of cell density, could favor QS over direct sensing (Box 2). Indeed, the co-regulation of stress responses by QS is a common theme (Lazazzera, 2000; Schuster and Greenberg, 2006; Mellbye and Schuster, 2010; Hense and Schuster, 2015). In *P. aeruginosa*, the overlap between the RpoS and QS regulons has been quantified by transcriptomics and includes dozens of genes (Schuster et al., 2004). For some co-regulated genes, both regulatory inputs, stress and density sensing, may be required to activate expression, whereas in other cases, one or the other may be sufficient.

Box 2 **Anticipating ecological conditions through QS.**

One explanation for the QS control of private goods is that cell density is a reliable predictor of environmental conditions. Hence, QS permits cell populations to anticipate and prepare for a given condition before it is actually encountered. Such anticipation may confer a distinct advantage to cells compared with a delayed response that is triggered by the direct sensing (DS) of the condition itself. Here we perform a simple cost-benefit analysis that compares the two regulatory strategies. We assume a scenario in which a private good is either regulated by QS or by DS, and we consider the degree to which QS anticipates correctly.

The net fitness benefit *F* of a given cell performing either QS or DS is then *F*_*QS*_ = *p* ^*^ *b*_*QS*_ – *c*_*QS*_, or *F*_*DS*_ = *b*_*DS*_ – *c*_*DS*_, with b and c as the respective costs and benefits, and *p* as the probability that QS-dependent expression provides a benefit at high cell density. We can then determine which strategy is favored as a function of relative costs, relative benefits, and the probability of a QS-dependent benefit (figure). The relative QS-dependent benefit is expressed as *b*_*QS*_/*b*_*DS*_ ≥1, because anticipation by QS is considered to provide a greater benefit than a delayed response by DS. The relative QS-dependent cost is expressed as *c*_*QS*_/*c*_*DS*_ ≤1, because the cost of QS regulation is considered to be smaller than the cost of DS regulation, which necessitates a dedicated regulatory pathway. It is assumed here that QS already has an established role in controlling other cellular functions and can simply be co-opted for this new purpose. The plane marks *F*_*QS*_ = *F*_*DS*_. Above the plane, QS is favored (*F*_*QS*_ > *F*_*DS*_), and below the plane, DS is favored (*F*_*QS*_ < *F*_*DS*_). Thus, the higher the relative cost of a dedicated DS pathway, and the greater the relative benefit from anticipation by QS, the lower the probability of a QS-dependent benefit can be to still favor QS regulation. In the case of phage infection, for example, the relative benefit of early, QS-dependent induction of resistance mechanisms may be so large (namely the difference between live and death) that QS regulation is worthwhile even if the predictive power of cell density is quite low.

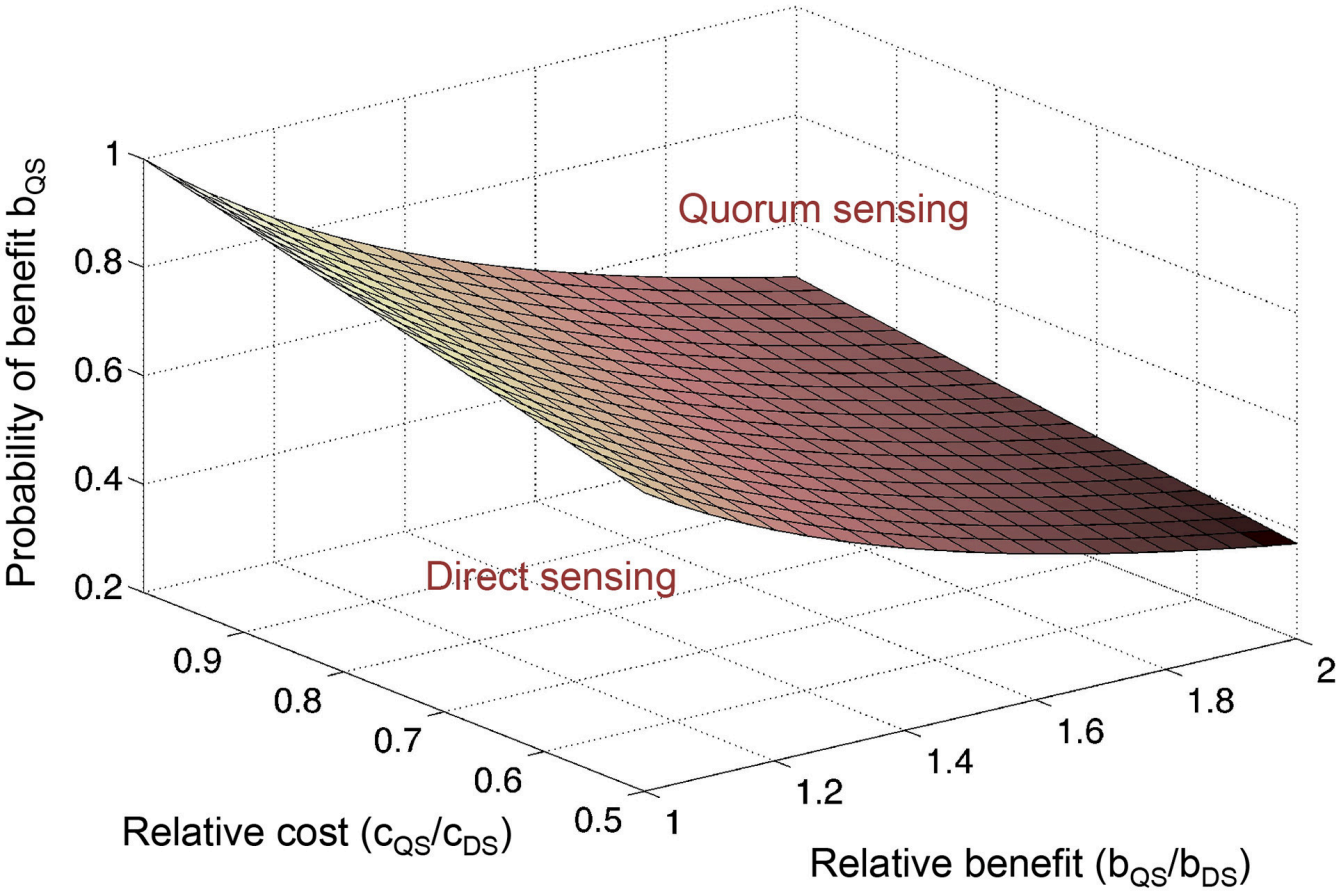

Among the many intracellular responses to stress are antibiotic resistance and oxidative stress resistance (Poole, 2012; Cornforth and Foster, 2013). In *P. aeruginosa*, a multi-drug RND efflux pump is co-regulated by QS and RpoS (Schuster et al., 2004), and two oxygen-detoxifying enzymes, superoxide dismutase and catalase, are regulated by QS (Hassett et al., 1999). In another opportunistic pathogen, *Providencia stewartii*, a cytoplasmic acetyltransferase that inactivates aminoglycoside antibiotics is regulated by QS (Ding et al., 2001). These types of QS-dependent functions can prepare bacterial pathogens not only for competition with other microbes but also for immune responses mounted by host cells. Host responses, including the production of reactive oxygen species or antimicrobial peptides, are more likely to be initiated once pathogens have reached high cell densities and attack the host via secretions that themselves may be, at least in part, controlled by QS (Miller and Britigan, 1997; Garcia-Contreras et al., 2015).

The QS control of contact-dependent secretion systems, such as Type VI secretion in *B. cepacia*, can also be interpreted in this context (Sana et al., 2012; Majerczyk et al., 2016). Functions include the toxins that are injected into and kill susceptible bacterial cells as well as the intracellular factors that provide immunity. We expect T6SS to be QS-controlled, because the proximity to competing cells is predicted to be closest in environments of high density (Cornforth and Foster, 2013). In addition, the usual, cell-density dependent benefit from secretion applies (even though the toxin is delivered into another cell rather than into the extracellular environment), because more cells benefit from the killing of competitors at high density.

If high cell density is a reliable predictor of nutrient exhaustion, then QS can also be expected to control appropriate metabolic adaptations. Indeed, in the plant pathogen *Burkholderia glumae*, a process described as “metabolic slowing” is thought to prepare cells for limited nutrient availability (An et al., 2014). It involves the QS-dependent decrease in glucose uptake, glycolysis, oxidative phosphorylation and nucleotide metabolism. In the vector-borne human pathogen *Yersinia pestis*, QS activates enzymes involved in maltose fermentation and the glyoxylate cycle (LaRock et al., 2013). These pathways may allow the use of alternative carbons sources available in a blood meal during growth in the flea. Upon exhaustion of the primary C-source, glucose, QS anticipates a switch to maltose and fatty acid utilization at high density.

Another type of biotic stress that is generally positively correlated with bacterial population density is the risk of phage infection (Levin and Bull, 2004; Figure 6B). Hence, the potential benefit of private anti-phage strategies increases with bacterial density. This property explains the QS-controlled repression of phage receptors in *Vibrio* species (Tan et al., 2015; Hoque et al., 2016), and induction of CRISPR-Cas phage immunity in *P. aeruginosa* (Hoyland-Kroghsbo et al., 2016).

Similarly, bacterial differentiation processes such as competence and sporulation are stress responses that are cell-density dependent and involve extensive changes in intracellular protein content (Claverys et al., 2006; Lopez and Kolter, 2010). Competence and the resulting genome innovation have the potential to improve adaptation to unfavorable environments, while the production of dormant spores increases the chances of survival. Here, too, regulation by QS may anticipate biotic stress at high cell density, although there are additional reasons that justify QS control (see Box 3).

Box 3 **Coordination of cell differentiation: sporulation and competence.**

QS signaling can be involved in the differentiation of a bacterial population into various cell types. A well-understood example is the differentiation process in *Bacillus subtilis* (Lopez and Kolter, 2010). Here, QS signals, together with other environmental cues, trigger differentiation into different phenotypes, including competence and sporulation. The underlying gene expression changes are generally bimodal and affect only a fraction of the population. This bimodality is often caused by stochastic differences in the expression levels of regulatory proteins that are amplified by positive autoregulation (Dubnau and Losick, 2006; Ackermann, 2015). Of course, differentiation into distinct, cooperating cell types only works if a critical number of cells exists. Such specialization is beneficial for two reasons, bet-hedging and division of labor (Ackermann, 2015). Some traits have obvious cooperative functions such as miners that secrete extracellular proteases to provide nutrients for the group (Veening et al., 2008). Others, notably competence and sporulation, involve intracellular processes and do not fit the standard description of cooperative behavior.

Genetic competence is defined as a state that permits the uptake of extracellular DNA, primarily for the purpose of transformation rather than nutrition (Johnston et al., 2014). The uptake machinery itself is composed of cellular membrane proteins and could be considered “private.” The benefit of DNA uptake and transformation, however, can be cell density-dependent. Competence involves the induction of bacteriocin systems directed at non-competent cells that do not express an immunity gene (Gonzalez-Pastor et al., 2003; Guiral et al., 2005). The killing efficiency of bacteriocin secretion increases with cell density, and so does the liberation of DNA for subsequent uptake by competent cells. From an inclusive fitness standpoint, genome innovation by a fraction of the population increases the chances of survival in fluctuating environments. Because the genetic alteration likely occurs at a locus other than that involved in competence, competence genes present in competent as well as non-competent cell types will be favored by natural selection.

A similar argument can be made for sporulation. Sporulation is the process of cellular differentiation into metabolically inactive spores that endure adverse environmental conditions (Piggot and Hilbert, 2004). The many products involved in the process of sporulation can be considered “private goods” as they are associated with the producing mother cell and provide no direct benefit to neighboring cells. Some non-sporulating cells lyse through the action of bacteriocins, as described above, and provide nutrients for the remainder of the population (Gonzalez-Pastor et al., 2003). This form of cannibalism has been interpreted as a mechanism that can delay the commitment to sporulation. However, it is also plausible that feeding on sister cells is more about ensuring that sporulation, an energetically costly process, can be completed. It is evident that this type of benefit attained from cannibalism is cell-density dependent.

Finally, QS can anticipate optimal group size and help maintain homeostasis in a colony or biofilm by controlling cell detachment (Kaplan, 2010). The QS-controlled factors responsible for this phenotype are, among others, biosurfactants, exoproteases, and flagella (Kaplan, 2010; Jang et al., 2014; Solano et al., 2014; Yang and Defoirdt, 2015). Biosurfactants and exoproteases are generally secreted, although in one case, the surfactant putisolvin produced by *P. putida* appears to be cell-associated and hence private (Carcamo-Oyarce et al., 2015). Flagella are also a private good, at least if they promote motility of an individual cell rather than groups of cells in coordinated swarms. In any case, dispersal can directly benefit the resident population due to its effects on group size: Whereas the beneficial effects of biofilm formation, such as protection from environmental stressors or protozoan grazing, are expected to saturate with group size, adverse effects such as starvation and accumulation of waste products are expected to increase (Aviles, 1999; Gjermansen et al., 2010). In addition, we may even consider the contribution of the dispersing cells to the inclusive fitness of resident cells. Inclusive fitness not only considers the fitness benefits of the focal individual, but also the benefits through other, related individuals that helps perpetuate shared genes (Hamilton, 1964; West et al., 2007b). Dispersal has the potential to colonize new habitat and thereby further increase the number of descendants.

### Metabolic changes associated with secretion

Cooperative secretion at high cell density can bring about significant changes in intracellular metabolism. We distinguish three categories. The first category, included here for completeness sake, deals with the most obvious functions of QS-controlled private goods, intracellular factors directly involved in the synthesis of, or protection from, a secreted product (Figure 6C). This category includes secretion systems for proteins and metabolites. Examples are the Type II protein secretion pathways in *P. aeruginosa* and in *Burkholderia glumae* that export numerous QS-controlled exoproteins (Chapon-Herve et al., 1997; Goo et al., 2010). This category further includes intracellular enzymes that produce an extracellular metabolite. Examples in *P. aeruginosa* are hydrogen cyanide synthase that produces the extracellular poison hydrogen cyanide and rhamnosyl transferase that produces the extracellular surfactant rhamnolipid (Pessi and Haas, 2000; Zhu and Rock, 2008). Another example in the plant pathogen *Pantoea stewartii* are enzymes of the *cps* biosynthetic pathway involved in the production of capsular polysaccharide (von Bodman et al., 1998). This category finally includes intracellular proteins that protect from self-produced secretions such as antibiotics and toxins. Examples include an alternative cytochrome c oxidase that protects from poisoning by self-produced cyanide in *P. aeruginosa* (Cunningham et al., 1997) and immunity factors of a toxin/antitoxin system in *Streptococcus mutans* (van der Ploeg, 2005).

The second category relates to changes in anabolic flux for secretion pathways that are further upstream from the actual secretion event and therefore less obvious compared to those just described. When QS activates the production of secreted factors, concomitant changes in primary metabolism are to be expected in order to provide the building blocks for synthesis reactions (Davenport et al., 2015; Goo et al., 2015; Hense and Schuster, 2015; Figure 6D). Hence, QS-control of private goods can have an accessory function in optimizing secretion, thereby increasing fitness. This will be particularly apparent for bacteria where QS controls an array of cooperative behaviors rather than an individual operon or phenotype (Schuster et al., 2003; Suppiger et al., 2013; Majerczyk et al., 2014; Ramachandran et al., 2014). *P. aeruginosa* again provides a good example. According to our previous transcriptome analysis, 162 out of 315 QS-activated genes in *P. aeruginosa* are annotated as “cytoplasmic” or “periplasmic” (Schuster et al., 2003; Gilbert et al., 2009). This analysis excludes obvious links to secretion, such as intracellular enzymes that synthesize extracellular metabolites or membrane-associated secretion machinery. A KEGG pathway analysis associates many of the 162 genes with metabolic functions, including carbon compound, central intermediary and energy metabolism (Table S1). A specific example is a number of metabolic enzymes involved in oxidative phosphorylation and in the Entner-Doudoroff pathway (Schuster et al., 2003; Heurlier et al., 2006), suggesting that QS adjusts aerobic utilization of certain carbon sources. Increased flux through this pathway could help provide the building blocks for the various carbon-rich secreted factors.

Another example is a set of genes connected with the metabolism of acyl-homoserine lactone (AHL) QS signals in *P. aeruginosa* (Table S1) (Schuster et al., 2003; Heurlier et al., 2006). Genes *glyA1* and *glyA2*, encoding serine hydroxymethyltransferases, and *gcvT2, gcvG2, gcvH2* encoding glycine cleavage system proteins, are activated by QS. These enzymes are involved in the synthesis of tetrahydrofolate, which serves as a methyl donor to homocysteine. Its methylation yields methionine, itself a precursor of the AHL substrate S-adenosyl methionine. Replenishment of the methionine pool may be required when AHL-signal synthase expression increases during QS activation. Heurlier et al. proposed that Nuh might also have role in AHL-metabolism by degrading the byproducts of synthesis reactions (Heurlier et al., 2006), although this cytoplasmic function seems inconsistent with the periplasmic location of Nuh.

The third category of metabolic changes encompasses catabolic adaptations to secretion (Figure 6E). Many QS-dependent secretions are, either directly or indirectly, involved in the acquisition of nutrients. Toxins damage other cells to release nutrients, and extracellular enzymes digest polymeric substrates to smaller forms suitable for uptake. As mentioned above, Nuh likely has a role in the salvaging of extracellular nucleosides made available by such QS-dependent secretions at high cell density. One might expect, then, that the QS-control of intracellular uptake and processing of nutrients made accessible by secreted products is a general phenomenon. These metabolic adaptations would essentially permit a cellular steady-state of simultaneous growth and secretion, in contrast to an alternation between both states as suggested above. Interestingly, however, QS-dependent intracellular nutrient processing does not appear to play a major role in the physiology of *P. aeruginosa*. A screening of more than 500 C and N sources revealed only a handful of compounds (adenosine, inosine, trehalose, and a few dipeptides) that a QS-proficient strain could utilize better than a QS mutant (Dandekar et al., 2012). It therefore appears that catabolic pathways in *P. aeruginosa* are largely directly regulated by the specific nutrient, without the involvement of QS. An apparent advantage of direct regulation is that the respective nutrients can be utilized whenever they are available, irrespective of cell density.

In general, a separation of secretion and cellular biomass synthesis is supported by the observation that parallel metabolic processes can cause biochemical conflicts in cells, favoring metabolic specialization (Ackermann, 2015). There could be either temporal or spatial specialization. The latter would permit a division of labor within a population of cells where some cells secrete while others catabolize and grow. This view is consistent with the finding that QS often only activates gene expression in a subpopulation of cells (Grote et al., 2015). Given the complexity of all these metabolic interactions, a systems-level approach, including metabolic modeling, will be beneficial for a deeper understanding of the roles of individual metabolic changes in QS-dependent adaptations.

### Leaky functions with private and public benefits

There is an increasing number of microbial functions whose benefits partition into private and public components (Morris et al., 2012; Estrela et al., 2016). The producing cell retains a fraction of the product, while the remaining fraction is either actively secreted or inevitably leaked into the extracellular environment. These products therefore constitute mixed goods. One example is a group of QS-controlled intracellular enzymes that affect the concentration of environmental substances, for example those related to oxidative stress, acidification, or antibiotic resistance (Hassett et al., 1999; Studer et al., 2008; Figure 6F). In these cases, the efficiency to reduce the local concentration of harmful environmental substances via intracellular enzymatic reactions increases if more cells contribute. Hence, a collective benefit to these activities is evident (Yurtsev et al., 2013). As discussed above, *P. aeruginosa* catalase, superoxide dismutase, and NADPH-producing dehydrogenases help remove reactive oxygen species (Hassett et al., 1999; Garcia-Contreras et al., 2015). *V. fischeri* acetyl coenzyme A synthetase helps resorb and catabolize extracellular acetate, preventing its accumulation to toxic levels (Studer et al., 2008), whereas *P. stuartii* acetyltransferase inactivates aminoglycoside antibiotics (Ding et al., 2001).

Other examples of leaky functions include nutrient scavenging molecules, such as yeast invertase (Gore et al., 2009) and bacterial siderophores (Imperi et al., 2009b; Kummerli et al., 2014; Scholz and Greenberg, 2015). Invertase and the *P. aeruginosa* siderophore pyoverdine are positively autoregulated in a way that represents a rudimentary form of QS. Invertase expression is stimulated by glucose, the product of invertase (Koschwanez et al., 2011), and pyoverdine expression is stimulated by iron-bound pyoverdine (Lamont et al., 2002). In both cases, cell density alters the concentration of a molecule that regulates gene expression. Invertase-dependent growth is cell-density dependent, as indicated above (Gore et al., 2009). Such information is available for the *E. coli* siderophore enterochelin rather than pyoverdine, although it is not known whether enterochelin expression is autoregulated. In this case, growth appears to be independent of cell density, presumably because the molecule is largely retained by the producing cell (Scholz and Greenberg, 2015). Nevertheless, analogous to yeast invertase, any good with a significant public component should provide a measurable cell-density dependent benefit and hence, justify regulation by QS.

Bacterial products particularly prone to inadvertent leakage are those located in the Gram-negative periplasm (Rinas and Hoffmann, 2004). Periplasmic leakage may be relevant to Nuh—the enzyme or its metabolites—from *P. aeruginosa* (Figure 6G). A previous study found no evidence for a density-dependent benefit of Nuh, but the initial cell densities chosen were quite high (Darch et al., 2012), conceivably far above the induction threshold for QS gene expression. We have previously shown that a QS mutant population can be invaded by the wild-type when grown on low concentrations of adenosine (Mellbye and Schuster, 2011), but this behavior does not necessarily imply that Nuh metabolites are not shared. As with yeast invertase, it only means that the behavior is not fully cooperative.

## Conclusions and implications

Here we have proposed different scenarios in which QS can serve to optimize a density-dependent benefit attained from private goods, providing an evolutionary “purpose” for their QS control. Taken together, we argue that that selection has brought traits under the control of QS whose benefits are associated with high cell-density environments (Popat et al., 2015). The association between benefit and cell density may be statistical (i.e., occur randomly but with certain probability, generally because relevant environmental conditions tend to correlate with cell density), or it may be deterministic (i.e., occur with certainty as a direct consequence of another action, generally because the private good itself is, directly or indirectly, linked to cooperation). Early anticipation of changes via QS adds a predictive element that may provide an advantage compared to a somewhat delayed response to the actual stimulus (Goo et al., 2012). For many traits, the optimal mode of control may be a combination of QS and other regulatory pathways. At low density, such co-regulation could mitigate some of the disadvantages from strict all-or-none QS-control. Cyanide resistance in *P. aeruginosa*, for example, is also directly induced by the presence of cyanide (Frangipani et al., 2014), which would even allow individual cells or a small group of cells to resist cyanide production by competing microbial species.

Using the ESS framework, we have also argued that QS control of private goods may not have evolved to stabilize public goods cooperation. This does not mean that pleiotropic cheater control does not happen or is not important; it only means that pleiotropic cheater control is a by-product for other benefits rather than an adaptation. It is important here to distinguish between the *optimization* and the *stabilization* of a behavior: The QS control of private goods has evolved because it optimizes a net benefit that is superior to other forms of regulation. The QS control of private goods is evolutionarily stable because of this optimization and because private goods - unlike public goods - preferentially benefit the producer. This includes products with a relatively small private share, as we have pointed out above.

The tractability of microbial model systems allows experimental verification of some of our predictions, in terms of both mechanism and evolutionary stability. Mechanisms can be tested according to the scenarios proposed above. As indicated, metabolic modeling could be useful in elucidating the role of central metabolism in optimizing secretion: Are the fluxes of central metabolites under quorate and non-quorate conditions in the model consistent with the experimentally observed regulation of the corresponding enzymes? Does the production of a given public good decrease when the fluxes through these reactions are restricted?

Evolutionary stability can be tested in co-culture competition and *in vitro* evolution experiments. Strains can be engineered such that they express a certain private good either constitutively or under QS control, and the fitness of the resulting strains can be compared. *In vitro* evolution under defined selective conditions can demonstrate the evolutionary stability of pleiotropic constraint and its susceptibility to invasion by mutant strategies. For example, using QS-controlled adenosine utilization in *P. aeruginosa* as a model, one could show that the QS control of Nuh breaks down and is replaced by constitutive expression if there is no cell density-dependent benefit to Nuh expression under the prevailing culture conditions.

Our analysis has implications for synthetic ecology (Dunham, 2007; Renda et al., 2014; Fredrickson, 2015). The propensity for cheating impedes the functionality of microbial communities engineered for the purpose of cooperative activities, for example biotechnological enzyme production. Our results suggest that the design of a regulatory constraint for the purpose of cheater control will have to be carefully considered. A mode of regulation that does not provide a fitness advantage over and could be invaded by an alternative mode of regulation would not be stable in the long-term.

Our analysis also has medical implications because the cooperative behaviors of many bacterial pathogens, such as QS, biofilm formation, or the secretion of toxins, are important virulence factors (Parsek and Singh, 2003; Rutherford and Bassler, 2012). Novel antivirulence strategies that target these behaviors have the potential to slow the evolution of resistance (Schuster et al., 2013; Allen et al., 2014; Ross-Gillespie et al., 2014). This beneficial effect is thought to be particularly relevant for cooperative behaviors because resistant cells would effectively behave as cooperators among a population of drug-sensitive cheaters in which cooperative virulence factor expression is suppressed (Andre and Godelle, 2005). This notion was confirmed experimentally by comparing the relative fitness of QS-proficient and QS-deficient strains of *P. aeruginosa* mimicking drug-resistant and drug-sensitive subpopulations, respectively (Mellbye and Schuster, 2011). QS-resistant mimics had an advantage only when growth was dependent on a cooperative rather than a non-cooperative trait under QS control. These results suggest that a clear functional analysis and distinction between public (cooperative) and private (non-cooperative) virulence factors is critical for treatment success.

Taken together, a deeper understanding of QS-controlled functions, and the evolutionary pressures that select for their maintenance, should improve our ability to predict evolutionary dynamics in natural environments, in pathogenic contexts, and in biotechnological applications.

## Author contributions

MS designed the study, evaluated the literature, analyzed data and wrote the manuscript. DS performed experiments, reviewed the literature, and analyzed data. BH designed the study, evaluated the literature, and wrote the manuscript.

### Conflict of interest statement

The authors declare that the research was conducted in the absence of any commercial or financial relationships that could be construed as a potential conflict of interest. The reviewer MTT declared a shared affiliation, though no other collaboration, with one of the authors BH to the handling Editor, who ensured that the process nevertheless met the standards of a fair and objective review.
