# Corrigendum: Why Quorum Sensing Controls Private Goods

**DOI:** 10.3389/fmicb.2017.01420

**Published:** 2017-07-24

**Authors:** Martin Schuster, D. Joseph Sexton, Burkhard A. Hense

**Affiliations:** ^1^Department of Microbiology, Oregon State University Corvallis, OR, United States; ^2^Institute of Computational Biology, Helmholtz Zentrum München Neuherberg, Germany

**Keywords:** quorum sensing, cooperation, cheating, public good, private good, evolutionary stability, *Pseudomonas aeruginosa*, nucleoside hydrolase

Here we intend to clarify the function of *rbsD*, a conserved gene involved in bacterial ribose utilization. As stated in the original article, *rbsD* is absent in *P. aeruginosa*, which is the likely cause for its slow growth rate on adenosine as a carbon source. Adenosine is cleaved into ribose and adenine by a periplasmic, quorum sensing-dependent nucleoside hydrolase (Nuh). In the section “The case of Nuh,” we suggested that *rbsD* contributes to ribose uptake, based on the original characterization of an *rbsD* mutant in *E. coli* (Oh et al., 1999). However, subsequent biochemical studies have revealed a more specific function. *E. coli rbsD* encodes a ribose mutarotase that catalyzes the conversion between the pyranose and furanose forms of D-ribose immediately after cytoplasmic uptake by the ribose transporter RbsABC (Kim et al., 2003; Ryu et al., 2004). While ribose primarily exists as a pyranose in solution, the furanose is the preferred substrate in the ensuing phosphorylation by the ribokinase RbsK (Sigrell et al., 1998). Thus, the intracellular level of the furanose as a substrate for RbsK may be the growth-limiting factor in *rbsD*-deficient *P. aeruginosa*.

Irrespective of these biochemical details, however, our main conclusions drawn in the original article remain the same: Adenosine is a relevant nitrogen but not carbon source in the ecology of *P. aeruginosa*. As a carbon source, adenosine does not constrain cheating in native *P. aeruginosa* but rather promotes non-social adaptation during long-term cultivation.

## Conflict of interest statement

The authors declare that the research was conducted in the absence of any commercial or financial relationships that could be construed as a potential conflict of interest.

## References

[B1] Kim M.-S. Shin J. Lee W. Lee H.-S. Oh B.-H. (2003). Crystal structure of RbsD leading to the identification of cytoplasmic sugar-binding proteins with a novel folding architecture. J. Biol. Chem. 278, 28173–28180. 10.1074/jbc.M304523200 12738765

[B2] Oh H. Park Y. Park C. (1999). A mutated PtsG, the glucose transporter, allows uptake of D-ribose. J. Biol. Chem. 274, 14006–14011. 10.1074/jbc.274.20.14006 10318813

[B3] Ryu K.-S. Changhoon Kim C. Kim I. Yoo S. Choi B.-S. Park C. (2004). NMR application probes a novel and ubiquitous family of enzymes that alter monosaccharide configuration. J. Biol. Chem. 279, 25544–25548. 10.1074/jbc.M402016200 15060078

[B4] Sigrell J. A. Cameron A. D. Jones T. A. Mowbray S. L. (1998). Structure of *Escherichia coli* ribokinase in complex with ribose and dinucleotide determined to 1.8 å resolution: insights into a new family of kinase structures. Structure 6, 183–193. 10.1016/S0969-2126(98)00020-3 9519409

